# Immune mediation between sarcopenia, performance status, and survival in gastric cancer patients undergoing radical resection

**DOI:** 10.3389/fonc.2025.1670034

**Published:** 2025-12-04

**Authors:** Pei Diao, Yan Wang, Xin Wang, Lijuan He

**Affiliations:** 1 Department of Physical Education, Neijiang Normal University, Neijiang, Sichuan, China; 2 Harbin Medical University Cancer Hospital, Harbin Medical University, Harbin, Heilongjiang, China; 3 Department of Gastrointestinal Surgery, The Affiliated Hospital, Southwest Medical University, Luzhou, Sichuan, China; 4 Department of Health Management Center, The Affiliated Hospital, Southwest Medical University, Luzhou, Sichuan, China

**Keywords:** gastric cancer, sarcopenia, immune status, prognosis, lymphocyte subsets

## Abstract

**Objective:**

This study aimed to explore the combined impact of sarcopenia and physical performance on immune status and prognosis in patients with resectable gastric cancer, and to evaluate the mediating role of immune function.

**Methods:**

A retrospective cohort of 413 patients undergoing radical gastrectomy was analyzed. Sarcopenia was assessed by skeletal muscle index (SMI) on CT, and physical performance by ECOG score. Patients were stratified into ISPS (Integrated Sarcopenia and Performance Status) high, medium, and low groups. Peripheral lymphocyte subsets were measured preoperatively. Kaplan–Meier curves, Cox regression, and mediation analysis were used to examine survival and immune interactions. Prognostic nomograms were constructed based on independent variables.

**Results:**

Lower ISPS scores were significantly associated with reduced CD3^+^, CD8^+^, and NK cells. Survival analysis showed that the ISPS-Low group had significantly worse progression-free survival (PFS, χ² = 27.36, P < 0.001) and overall survival (OS, χ² = 31.54, P < 0.001). ISPS and CD8^+^ T cell levels were independent predictors of both PFS and OS. Mediation analysis indicated that CD8^+^ T cells partially mediated the effect of ISPS on survival, accounting for approximately 24% of the total effect. Nomograms incorporating ISPS, immune status, TNM stage, and tumor size demonstrated good predictive performance (C-index: 0.732 for PFS, 0.718 for OS).

**Conclusion:**

Sarcopenia and poor functional status are linked to impaired immunity and unfavorable outcomes in gastric cancer. Immune status may partially explain this relationship. ISPS may serve as a useful prognostic tool, and integrating physical and immune assessments could improve prognostic evaluation and support personalized perioperative strategies.

## 1 Introduction

Gastric cancer remains one of the most common and deadly malignancies worldwide, particularly in East Asian countries such as China, Japan, and Korea (1). According to the latest global cancer statistics, gastric cancer ranks fifth in incidence and fourth in cancer-related mortality, posing a significant public health burden (2). Despite advances in diagnostic techniques and therapeutic strategies, radical gastrectomy combined with perioperative chemotherapy remains the cornerstone of curative treatment for resectable gastric cancer (3). However, the aggressive nature of both the disease and its treatment often results in profound nutritional deterioration. Gastric resection can significantly impair digestive and absorptive functions, while tumor-associated systemic inflammation and metabolic dysregulation further exacerbate malnutrition, sarcopenia, and functional impairment (4). These nutritional and functional impairments not only compromise treatment tolerance but are increasingly recognized as critical determinants of immune status and long-term prognosis. Therefore, understanding the impact of functional impairment and sarcopenia on immune status and oncologic outcomes is essential for optimizing perioperative care and improving survival in patients undergoing radical resection for gastric cancer.

Sarcopenia, defined as the progressive loss of skeletal muscle mass and function, is common yet often overlooked in patients with gastrointestinal malignancies (5). In the perioperative setting, it is associated with higher rates of postoperative complications, delayed recovery, prolonged hospitalization, and poorer long-term survival (6). The skeletal muscle index (SMI), calculated from computed tomography (CT) images at the third lumbar vertebral level, is a widely accepted and objective measure of muscle mass (7). Its accuracy, reproducibility, and accessibility from routine preoperative imaging make it attractive in clinical practice (8). However, SMI reflects muscle quantity but not functional capacity, such as strength, endurance, or daily activity. These limitations highlight the need for complementary approaches to assess functional impairment more comprehensively, which may better inform prognosis and treatment tolerance.

To bridge structure and function, we integrated the complementary information captured by SMI (muscle mass) and Eastern Cooperative Oncology Group (ECOG, functional capacity) into a single metric (ISPS). These domains are related but non-redundant. Combining them is expected to reflect overall physiologic reserve more faithfully than either measure alone. We therefore prespecified ISPS by coupling low SMI with functional impairment and hypothesized an association with reduced peripheral CD8^+^ T-cell levels and poorer survival. To complement mass-based assessments such as SMI, ECOG performance status is widely used to assess functional capacity and treatment fitness (9). Higher ECOG scores, indicating functional impairment, are independently associated with inferior survival across malignancies (10–12). Sarcopenia and functional impairment often coexist as markers of reduced physiologic reserve (13), which compromise treatment tolerance and may impair immune function, thereby influencing disease progression and prognosis. Compared with SMI or ECOG alone, the composite ISPS captures both structural and functional dimensions, thereby providing stronger prognostic discrimination and mechanistic plausibility as it reflects overall physiologic reserve.

Extensive research has established that the host immune response plays a central role in cancer progression, recurrence, and treatment outcomes (14–16). In gastrointestinal cancers, sarcopenia or frailty is directly linked to systemic immune dysregulation, with patients commonly showing altered peripheral lymphocyte subsets and elevated inflammatory indices (e.g., NLR, SII, SIRI), which correlate with inferior survival (17). In gastric cancer specifically, sarcopenia or myosteatosis predicts poorer outcomes with immune checkpoint inhibitors, and combining lymphocyte subpopulations with muscle-quality metrics improves prognostic stratification. Moreover, musculoskeletal deficits such as osteosarcopenia associate with alterations in the tumor microenvironment in advanced gastric cancer (18). In gastric cancer, systemic immune dysfunction, characterized by reduced lymphocyte counts, impaired cytotoxic T-cell function, and elevated inflammatory markers, is associated with poor prognosis (19). Sarcopenia and functional impairment may contribute to this state by inducing chronic inflammation, altering metabolic balance, and reducing physical resilience (20). These factors collectively undermine the body’s ability to sustain an effective antitumor immune response. Peripheral blood lymphocyte subsets provide a convenient, reproducible, and minimally invasive measure of systemic immune status (21). Therefore, integrating immune profiling with physical and nutritional assessments may enhance prognostic evaluation and guide personalized perioperative management (22). Few studies, however, have examined the combined effects of sarcopenia, functional impairment, and peripheral immune status in resectable gastric cancer. Immune dysfunction may also plausibly act as a mediator linking sarcopenia to adverse oncologic outcomes, although this pathway has rarely been explicitly examined in gastric cancer.

Given the growing recognition of the prognostic significance of physical condition and immune status in gastric cancer, there remains a critical need to clarify how sarcopenia and functional impairment together influence systemic immunity and long-term clinical outcomes. Most existing studies have evaluated these factors independently, without integrating comprehensive assessments of muscle mass, functional status, and peripheral immune parameters. To address this gap, the present study aimed to investigate the combined effects of sarcopenia and functional impairment on immune status, as reflected by peripheral blood lymphocyte subsets, and on overall survival in patients with resectable gastric cancer undergoing radical gastrectomy. Furthermore, we explored whether immune status mediates the relationship between physical function and prognosis, in order to better understand the underlying mechanisms linking physiologic reserve to oncologic outcomes. By incorporating structural, functional, and immunological indicators, this study seeks to establish a more holistic framework for perioperative risk stratification and individualized patient management.

## 2 Patients and methods

### 2.1 Patients

This retrospective cohort study was conducted at the Affiliated Hospital of Southwest Medical University (Luzhou, China). Patients who underwent radical resection for gastric adenocarcinoma between January 2019 and December 2023 were screened for eligibility. All patient data were retrieved from the hospital’s electronic medical record system.

The inclusion criteria were as follows: (i) age ≥18 years; (ii) histologically confirmed gastric cancer; (iii) availability of a preoperative abdominal computed tomography (CT) scan performed within one month prior to surgery for SMI assessment; (iv) availability of a peripheral blood sample collected within 7 days before surgery and analyzed using flow cytometry for immune profiling; and (v) complete clinical, pathological, and follow-up data. Patients were excluded if they met any of the following criteria: (i) received neoadjuvant chemotherapy or radiotherapy prior to surgery; (ii) had a diagnosis of another malignancy or evidence of distant metastasis at the time of surgery; (iii) had a history of autoimmune disease or were receiving long-term immunosuppressive therapy; or (iv) had incomplete clinical information or missing imaging or immune profiling data.

**Figure 1:**
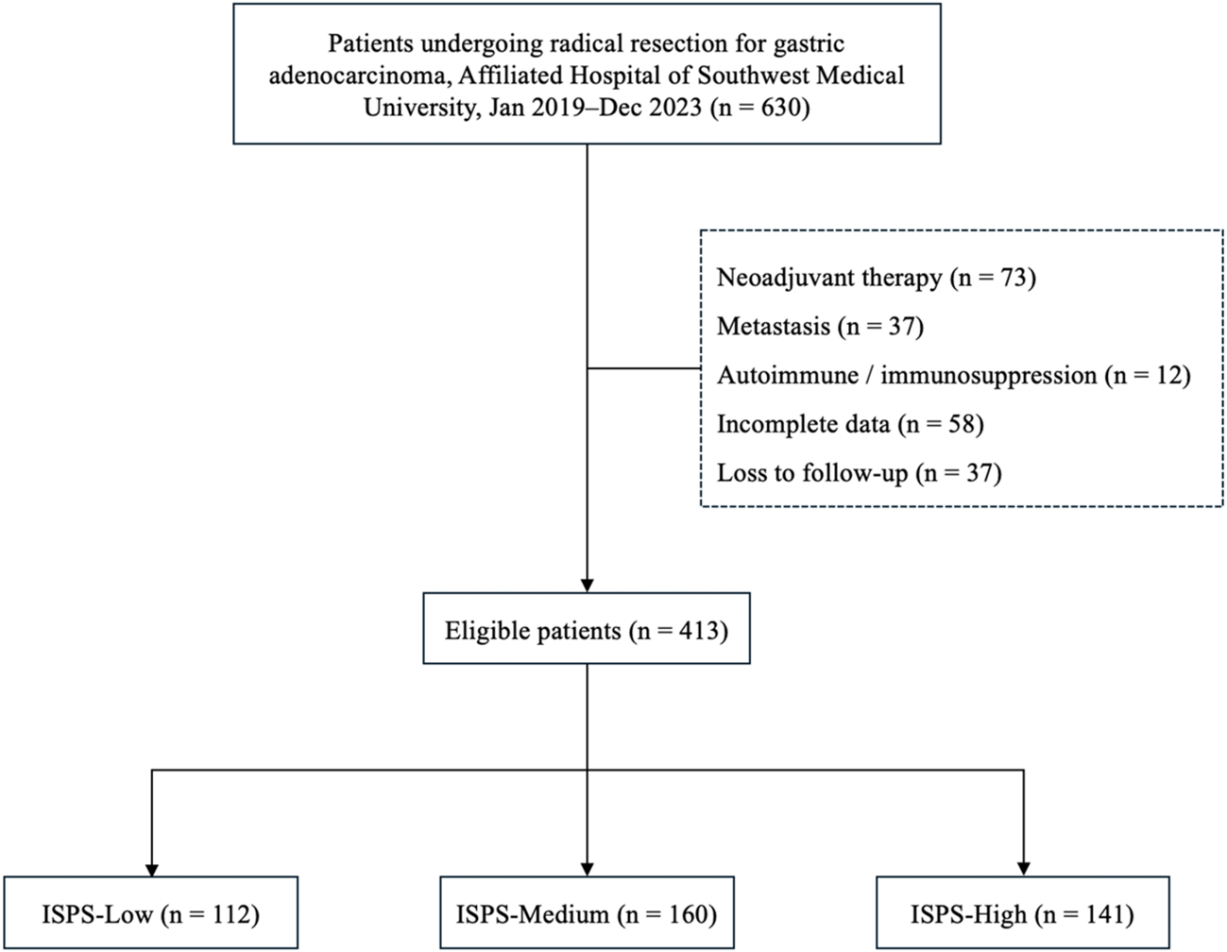
Flowchart of patient selection and grouping.

A total of 413 eligible patients were included in the final analysis. The study was approved by the Ethics Committee of the Affiliated Hospital of Southwest Medical University (Approval No. KY2025353). All procedures were performed in accordance with the ethical standards of the Declaration of Helsinki and its subsequent amendments. The requirement for written informed consent was waived due to the retrospective and anonymized nature of the study. The patient selection process is summarized in **Figure 1**.

### 2.2 Assessment of sarcopenia

**Figure 2:**
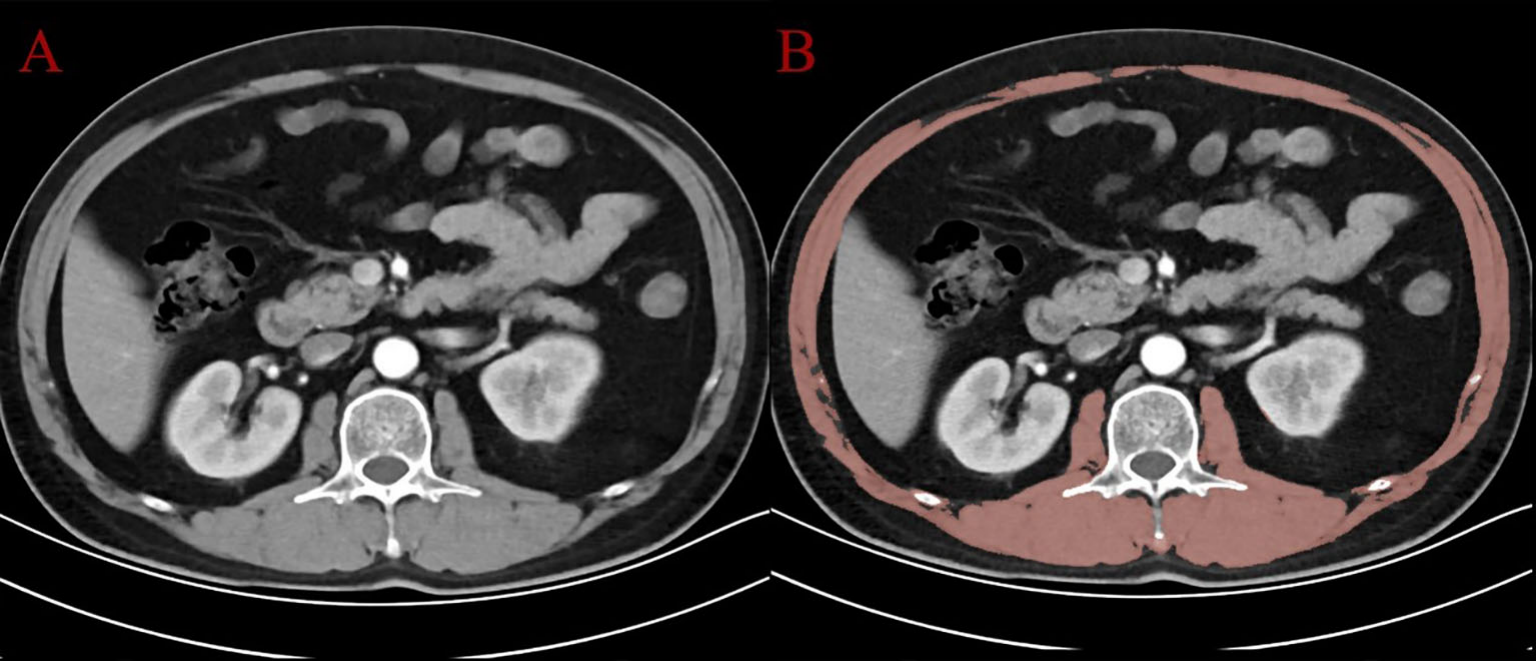
CT image at the L3 level: **(A)** original axial slice; **(B)** segmented skeletal muscle (in red) using 3D Slicer.

Sarcopenia was evaluated based on the skeletal muscle area (SMA) measured at the level of the third lumbar vertebra (L3) on axial CT images. Using 3D Slicer software (version 5.8.1), the axial CT slice corresponding to the L3 vertebral level was identified for each patient. A segmentation procedure was performed to delineate skeletal muscle tissue, including the psoas major, erector spinae, quadratus lumborum, and abdominal wall muscles (external oblique, internal oblique, and transversus abdominis). The segmentation process combined semi-automated thresholding (Hounsfield units ranging from -29 to +150) with manual correction using the “Paint” and “Erase” tools in the Segment Editor module. Non-muscle structures such as intra-abdominal organs and subcutaneous adipose tissue were carefully excluded. **Figure 2** shows an example of the original CT image (Panel A) and the segmented muscle area (Panel B). After segmentation, the cross-sectional area (cm²) of the skeletal muscle was automatically calculated by the software. SMI was then derived by normalizing the SMA to the square of the patient’s height (cm²/m²). The diagnostic cutoff values for sarcopenia were set at SMI < 52.4 cm²/m² for men and < 38.5 cm²/m² for women, according to previously validated criteria (23).

### 2.3 Assessment of physical function and ISPS classification

Preoperative physical function was assessed using ECOG, a standardized and widely accepted tool for evaluating a patient’s level of physical activity, independence, and ability to tolerate medical treatment. The ECOG scale ranges from 0 to 5, with higher scores indicating greater functional impairment. A score of 0 denotes full activity without limitations; 1 reflects minor restrictions in strenuous activity; 2 indicates the patient is ambulatory and capable of self-care but unable to carry out work-related activities; 3 represents limited self-care and confinement to bed or chair for more than 50% of waking hours; and 4 signifies complete disability. In this study, ECOG PS was obtained from preoperative medical records, and patients were categorized into two groups: good physical function (ECOG 0-1) and impaired physical function (ECOG ≥2).

To capture the combined impact of skeletal muscle mass and functional status, we developed ISPS classification system. This composite index reflects physiological vulnerability by combining two clinically important parameters, namely sarcopenia defined by skeletal muscle index thresholds and ECOG performance status. Patients were classified into three ISPS groups based on their structural and functional reserves. The ISPS-High group included patients without sarcopenia and with an ECOG PS of 0-1, indicating preserved muscle mass and good functional capacity. The ISPS-Medium group comprised individuals with either sarcopenia or an ECOG PS of ≥2, but not both, reflecting a moderate degree of impairment in either domain. The ISPS-Low group included patients who presented with both sarcopenia and an ECOG PS of ≥2, representing a severely compromised physical status.

### 2.4 Flow cytometry and immune cell profiling

**Figure 3:**
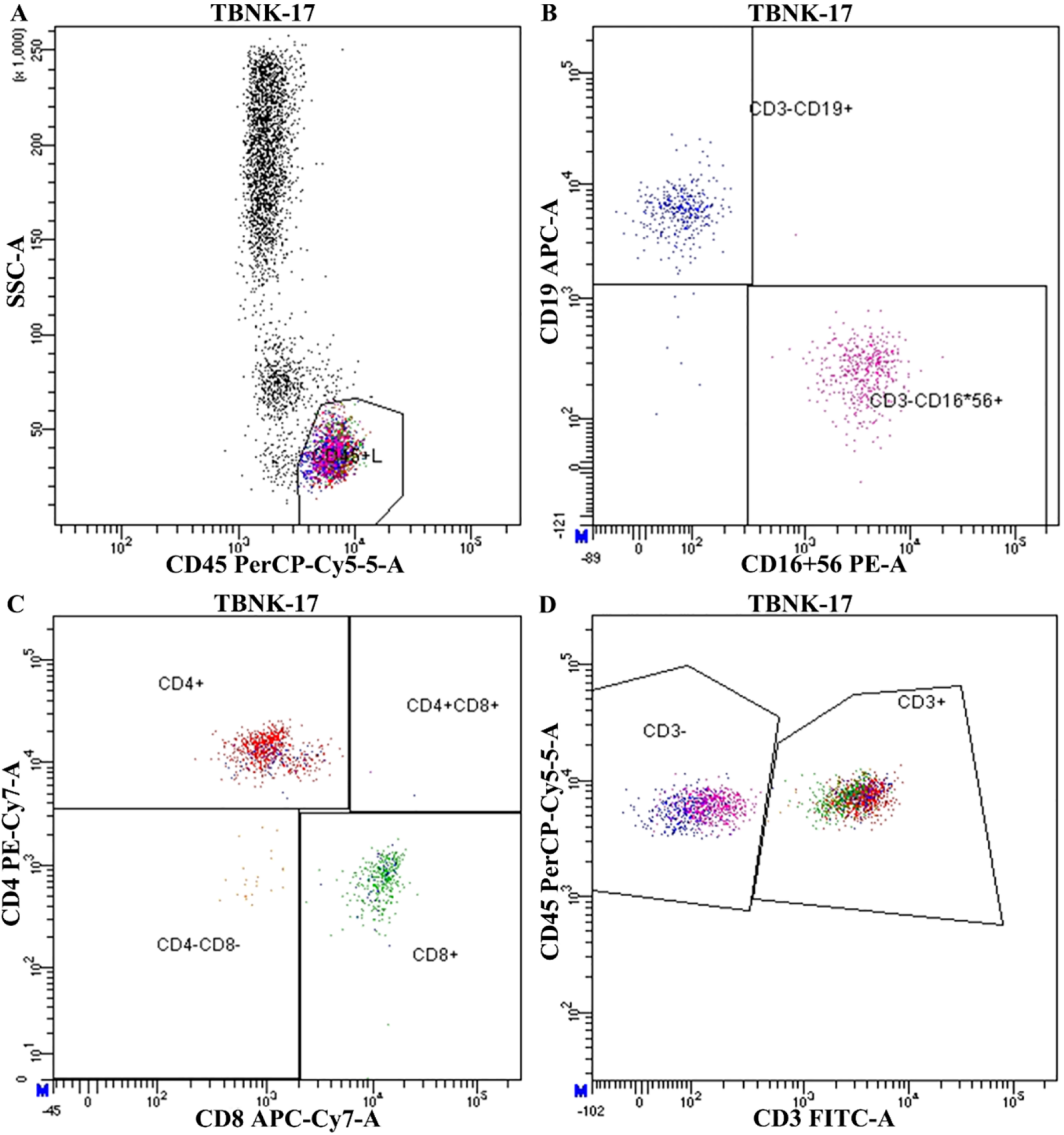
Representative flow cytometry gating strategy for peripheral blood lymphocyte subset analysis. **(A)** Initial gating of lymphocyte population using side scatter (SSC-A) vs CD45 PerCP-Cy5.5. **(B)** Identification of CD19^+^ B cells and CD16^+^56^+^ NK cells within the CD3^−^ population. **(C)** Subtyping of T cells based on CD4 and CD8 expression. **(D)** Identification of total CD3^+^ T lymphocytes.

Peripheral blood samples were collected within 1–2 days prior to surgery for immunophenotyping analysis. Whole blood was processed using a standardized TBNK (T cells, B cells, and NK cells) panel. Fluorochrome-conjugated monoclonal antibodies against CD3, CD4, CD8, CD19, CD16, CD56, and CD45 were used to identify lymphocyte subsets. Flow cytometry was performed on a BD FACSCanto II flow cytometer (BD Biosciences, USA), and data were analyzed using FlowJo software (version 10.8.1, TreeStar, USA). The gating strategy involved initial identification of lymphocytes based on side scatter (SSC-A) and CD45 expression. CD3^+^ T cells were then separated from CD3^−^ cells, among which CD19^+^ B cells and CD16^+^56^+^ NK cells were further identified. Within the CD3^+^ population, T cell subsets were analyzed based on CD4 and CD8 expression, yielding four major subgroups: CD4^+^, CD8^+^, CD4^+^CD8^+^, and CD4^−^CD8^−^ cells. A representative gating strategy is shown in **Figure 3**. Immune cell subset proportions were calculated as percentages of total lymphocytes and were used for downstream statistical analysis.

### 2.5 Data collection and follow-up

Clinical data were retrospectively extracted from the electronic medical records of all 413 eligible patients. Baseline characteristics included demographic variables (age, sex, and BMI), tumor-related parameters (tumor location, maximum tumor diameter, histological differentiation, Lauren classification, and pathological TNM stage), physical status (ECOG performance score and sarcopenia status), and immune profiling results obtained from flow cytometry.

Follow-up data were obtained through outpatient visits, telephone interviews, and review of hospital records. Patients were evaluated every 3 to 6 months during the first 2 years after surgery and annually thereafter. The final follow-up was completed in March 2024. The median follow-up duration for the entire cohort was 42.6 months (range, 3.0 to 78.5 months). Progression-free survival (PFS) was defined as the interval from the date of radical gastrectomy to the first documented recurrence, disease progression, or death from any cause, whichever occurred first. Overall survival (OS) was defined as the time from surgery to death from any cause or the date of last follow-up. Patients lost to follow-up were censored at the date of last confirmed contact.

### 2.6 Statistical analysis

All statistical analyses were performed using R software (version 4.3.2) and SPSS (version 26.0). The distribution of continuous variables was assessed using the Shapiro–Wilk test. Normally distributed data were expressed as mean ± standard deviation (SD), while non-normally distributed data were presented as median and interquartile range (IQR). Between-group comparisons were conducted using independent-samples t-test or one-way ANOVA for normally distributed variables, and the Mann–Whitney U test for non-normally distributed variables. Categorical variables were expressed as frequencies and percentages and compared using the chi-square test or Fisher’s exact test, as appropriate. To evaluate differences in survival outcomes, Kaplan–Meier survival curves were generated, and the log-rank test was used to compare survival between groups. To reduce potential baseline confounding, propensity score matching (PSM) was performed based on relevant baseline covariates. A 1:1 nearest-neighbor matching algorithm was applied with a caliper width of 0.02. Baseline balance between matched groups was assessed using standardized mean difference (SMD), with an SMD < 0.1 considered indicative of acceptable covariate balance. Univariate Cox proportional hazards models were used to identify potential prognostic factors for PFS and OS. Variables with P < 0.05 in univariate analysis were included in multivariate Cox regression to identify independent prognostic indicators. Hazard ratio (HR) and 95% confidence interval (CI) were reported. Subgroup analyses were further performed stratified by BMI and TNM stage, and interaction tests were used to examine the consistency of effects across strata. To develop individualized survival prediction tools, nomograms were constructed for both PFS and OS based on the final multivariate Cox models. The predictive accuracy of the nomograms was assessed using calibration curves and C-index. In addition, a mediation analysis was conducted to investigate whether specific intermediate variables could account for the relationship between clinical composite indices and survival outcomes. A structural equation modeling (SEM) framework was applied to estimate total, direct, and indirect effects. Statistical significance and 95% confidence intervals for indirect effects were obtained using bootstrap resampling with 5,000 iterations. Finally, a *post-hoc* sample size and power evaluation was performed using events-per-variable (EPV) calculation and the Schoenfeld approximation. All statistical tests were two-sided, and a P *< 0.05* was considered statistically significant.

## 3 Results

### 3.1 Patients characteristics

A total of 413 patients who underwent radical resection for gastric cancer were included in this study, with a median age of 55.8 years. Among them, 309 (74.8%) were male and 104 (25.2%) were female. According to the ISPS classification system, patients were categorized into three groups: ISPS-Low (n=112), ISPS-Medium (n=160), and ISPS-High (n=141). The detailed clinicopathological characteristics of each group are summarized in **Table 1**. Significant differences were observed among the three groups in terms of age (P<0.001) and body mass index (BMI, P<0.001), with patients in the high ISPS group tending to be younger and having a higher BMI. No significant differences were found in sex distribution across the groups (P = 0.311). However, smaller tumors (<20 mm) were more frequently observed in the high ISPS group, whereas larger tumors (>50 mm) were more common in the low ISPS group (P = 0.021). Tumor differentiation also varied significantly among the groups (P = 0.047), with a higher proportion of well-differentiated tumors observed in the high ISPS group. Furthermore, a strong correlation was found between the ISPS classification and TNM stage (P<0.001). Specifically, 37 patients (9.0%) were classified as stage I, 135 (32.7%) as stage II, and 241 (58.4%) as stage III. Early-stage disease (stage I–II) was more prevalent in the high ISPS group, while advanced-stage disease (stage III) was more common in the low ISPS (74.1%). These findings suggest that a higher ISPS score may be associated with more favorable clinicopathological features, highlighting its potential prognostic value in gastric cancer.

**Table 1:** Patients characteristics.

Items	ISPS-Low	ISPS-Medium	ISPS-High	*P* value
	n=112	n=160	n=141	
Age (years), mean (SD)	58.6 ± 7.8	54.4 ± 8.2	52.3 ± 8.9	<0.001
Sex (%)				
Male	84 (75.0)	115 (72.0)	110 (78.0)	0.311
Female	28 (25.0)	45 (28.0)	31 (22.0)	
BMI (Kg/m^2^), mean (SD)	19.2 ± 2.4	20.4 ± 2.1	21.7 ± 2.0	<0.001
Primary tumor site (%)				0.285
Upper 1/3	16 (14.3)	24 (13.8)	19 (14.0)	
Middle 1/3	34 (30.4)	48 (27.6)	44 (32.4)	
Low 1/3	45 (40.2)	64 (36.8)	58 (42.6)	
Whole	17 (15.2)	24 (13.8)	20 (14.7)	
Tumor size (%)				0.021
<20 mm	15 (13.4)	23 (13.2)	29 (21.3)	
20–50 mm	56 (50.0)	86 (49.4)	73 (53.7)	
>50 mm	41 (36.6)	51 (29.3)	39 (28.7)	
Differentiation (%)				0.047
Poor	54 (48.2)	73 (42.0)	53 (39.0)	
Moderately	31 (27.7)	48 (27.6)	48 (35.3)	
Well	11 (9.8)	21 (12.1)	23 (16.9)	
Unknown	16 (14.3)	18 (10.3)	17 (12.5)	
Lauren type (%)				0.116
Intestinal	42 (37.5)	63 (36.2)	77 (56.6)	
Diffuse	54 (48.2)	74 (42.5)	48 (35.3)	
Mixed	9 (8.0)	13 (7.5)	10 (7.4)	
Unknown	7 (6.2)	10 (5.7)	6 (4.4)	
TNM stage (%)				<0.001
I	5 (4.5)	12 (6.9)	20 (14.7)	
II	24 (21.4)	50 (28.7)	61 (44.9)	
III	83 (74.1)	98 (56.3)	60 (44.1)	

### 3.2 Association between ISPS and peripheral lymphocyte subsets

To investigate the relationship between physical status and immune function, we compared the distributions of peripheral blood lymphocyte subsets across different ISPS groups (**Table 2**). Significant differences were observed in the percentages of CD3^+^, CD8^+^, and CD3^−^CD16^+^CD56^+^ lymphocytes. The proportion of total T cells (CD3^+^) increased progressively with better ISPS classification: 63.5% ± 4.1% in the ISPS-Low group, 67.4% ± 3.8% in the ISPS-Medium group, and 72.9% ± 4.0% in the ISPS-High group (P = 0.001). Similarly, the percentage of cytotoxic T cells (CD8^+^) was significantly higher in patients with better functional and structural status, rising from 22.5% ± 2.5% in ISPS-Low to 29.8% ± 2.7% in ISPS-High (P < 0.001). A comparable pattern was found in the NK cell population (CD3^−^CD16^+^CD56^+^), with a statistically significant increase across ISPS groups (1.62% ± 0.7% vs. 2.11% ± 0.8% vs. 2.14% ± 0.9%, P = 0.005). However, no significant differences were observed in the proportions of helper T cells (CD4^+^), double-positive T cells (CD4^+^CD8^+^), or B cells (CD19^+^) among the three groups (all P > 0.05). These findings suggest that patients with preserved muscle mass and physical function (higher ISPS) tend to exhibit enhanced systemic immune activity, particularly reflected in elevated CD3^+^, CD8^+^, and NK cell levels.

**Table 2:** Lymphocyte subsets according to ISPS groups.

Items	ISPS-Low	ISPS-Medium	ISPS-High	*P* value
	n=112	n=160	n=141	
CD3^+^ (%)	63.5 ± 4.1	67.4 ± 3.8	72.9 ± 4.0	0.001
CD4^+^ (%)	40.2 ± 3.2	40.1 ± 3.1	41.3 ± 3.4	0.213
CD8^+^ (%)	22.5 ± 2.5	25.3 ± 2.4	29.8 ± 2.7	<0.001
CD3^+^CD4^+^CD8^+^ (%)	11.2 ± 3.0	11.7 ± 2.8	11.1 ± 2.9	0.629
CD19^+^ (%)	15.0 ± 3.2	15.5 ± 3.0	15.1 ± 3.4	0.442
CD3^-^CD16^+^CD56^+^ (%)	1.62 ± 0.7	2.11 ± 0.8	2.14 ± 0.9	0.005

### 3.3 Univariate and multivariate Cox analysis

To identify prognostic factors associated with survival outcomes, both univariate and multivariate Cox analyses were performed for PFS and OS. Univariate analysis identified several variables significantly associated with PFS, including age (P = 0.041), BMI (P = 0.034), ISPS (P < 0.001), tumor size (P = 0.017), TNM stage (P < 0.001), CD3^+^ T cells (P = 0.045), CD8^+^ T cells (P < 0.001), and CD19^+^ B cells (P = 0.037). Multivariate Cox regression analysis was performed by including variables with P < 0.05 in the univariate analysis. Before performing multivariate Cox regression, multicollinearity among the included covariates was examined. As shown in **Table 2**, all variables had tolerance values >0.6 and variance inflation factors (VIFs) <2, indicating that no significant multicollinearity was present (**Table 3**). The results indicated that ISPS (HR = 0.631, 95% CI: 0.432–0.900, P < 0.001), TNM stage (HR = 3.655, 95% CI: 2.263–4.309, P < 0.001), and CD8^+^ T cell proportion (HR = 0.892, 95% CI: 0.831–0.964, P = 0.005) remained independent prognostic factors for PFS (**Table 4**).

**Table 3:** The tolerance and VIF of all burnout related factors.

Items	Tolerance	VIF
Age	0.734	1.362
BMI	0.812	1.232
ISPS score	0.953	1.049
Tumor size	0.877	1.141
TNM stage	0.698	1.433
CD3^+^	0.765	1.307
CD8^+^	0.921	1.086
CD19^+^	0.846	1.182

**Table 4:** Cox regression analysis for PFS.

Parameters	PFS			
	Univariate analysis	*P*	Multivariate analysis	*P*
	HR (95% CI)		HR (95% CI)	
Age	1.022 (1.001–1.044)	0.041	1.002 (0.988–1.054)	0.231
Sex	1.084 (0.725–1.619)	0.659		
BMI	0.942 (0.789–0.998)	0.034	0.987 (0.889–1.003)	0.449
ISPS score	0.566 (0.388–0.828)	<0.001	0.631 (0.432–0.900)	<0.001
Primary tumor site	1.118 (0.834–1.415)	0.584		
Tumor size	1.313 (1.075–1.648)	0.017	1.152 (0.771–1.429)	0.346
Differentiation	1.115 (0.887–1.417)	0.374		
Lauren type	1.104 (0.833–1.304)	0.243		
TNM stage	4.248 (3.447–4.985)	<0.001	3.655 (2.263–4.309)	<0.001
CD3^+^	0.978 (0.956–0.998)	0.045	0.989 (0.965–1.010)	0.123
CD4^+^	0.980 (0.951–1.016)	0.194		
CD8^+^	0.852 (0.820–0.914)	<0.001	0.892 (0.831–0.964)	0.005
CD3^+^CD4^+^CD8^+^	1.002 (0.943–1.066)	0.928		
CD19^+^	0.970 (0.886–0.998)	0.037	0.998 (0.922–1.019)	0.589
CD3^-^CD16^+^CD56^+^	0.953 (0.844–1.082)	0.435		

For OS, univariate Cox regression analysis showed that ISPS (P < 0.001), tumor size (P = 0.009), TNM stage (P < 0.001), and CD8^+^ T cell proportion (P < 0.001) were significantly correlated with OS. In the multivariate Cox regression model, ISPS (HR = 0.645, 95% CI: 0.468–0.889, P = 0.007), TNM stage (HR = 3.305, 95% CI: 2.119–4.121, P < 0.001), tumor size (HR = 1.273, 95% CI: 1.065–1.578, P = 0.012), and CD8^+^ T cell proportion (HR = 0.903, 95% CI: 0.846–0.974, P = 0.006) were identified as independent prognostic factors for OS (**Table 5**).

**Table 5:** Cox regression analysis for OS.

Parameters	PFS			
	Univariate analysis	*P*	Multivariate analysis	*P*
	HR (95% CI)		HR (95% CI)	
Age	1.018 (0.997–1.039)	0.093		
Sex	1.062 (0.701–1.608)	0.773		
BMI	0.951 (0.879–1.042)	0.221		
ISPS score	0.579 (0.410–0.798)	<0.001	0.645 (0.468–0.889)	0.007
Primary tumor site	1.127 (0.850–1.395)	0.482		
Tumor size	1.302 (1.082–1.613)	0.009	1.273 (1.065–1.578)	0.012
Differentiation	1.099 (0.871–1.375)	0.442		
Lauren type	1.088 (0.827–1.290)	0.278		
TNM stage	3.969 (3.111–4.738)	<0.001	3.305 (2.119–4.121)	<0.001
CD3^+^	0.981 (0.960–1.008)	0.139		
CD4^+^	0.983 (0.951–1.020)	0.315		
CD8^+^	0.861 (0.831–0.918)	<0.001	0.903 (0.846–0.974)	0.006
CD3^+^CD4^+^CD8^+^	1.008 (0.954–1.072)	0.716		
CD19^+^	0.968 (0.897–1.012)	0.198		
CD3^+^CD16^+^CD56^+^	0.958 (0.856–1.093)	0.488		

### 3.4 Subgroup and interaction analysis

**Figure 4:**
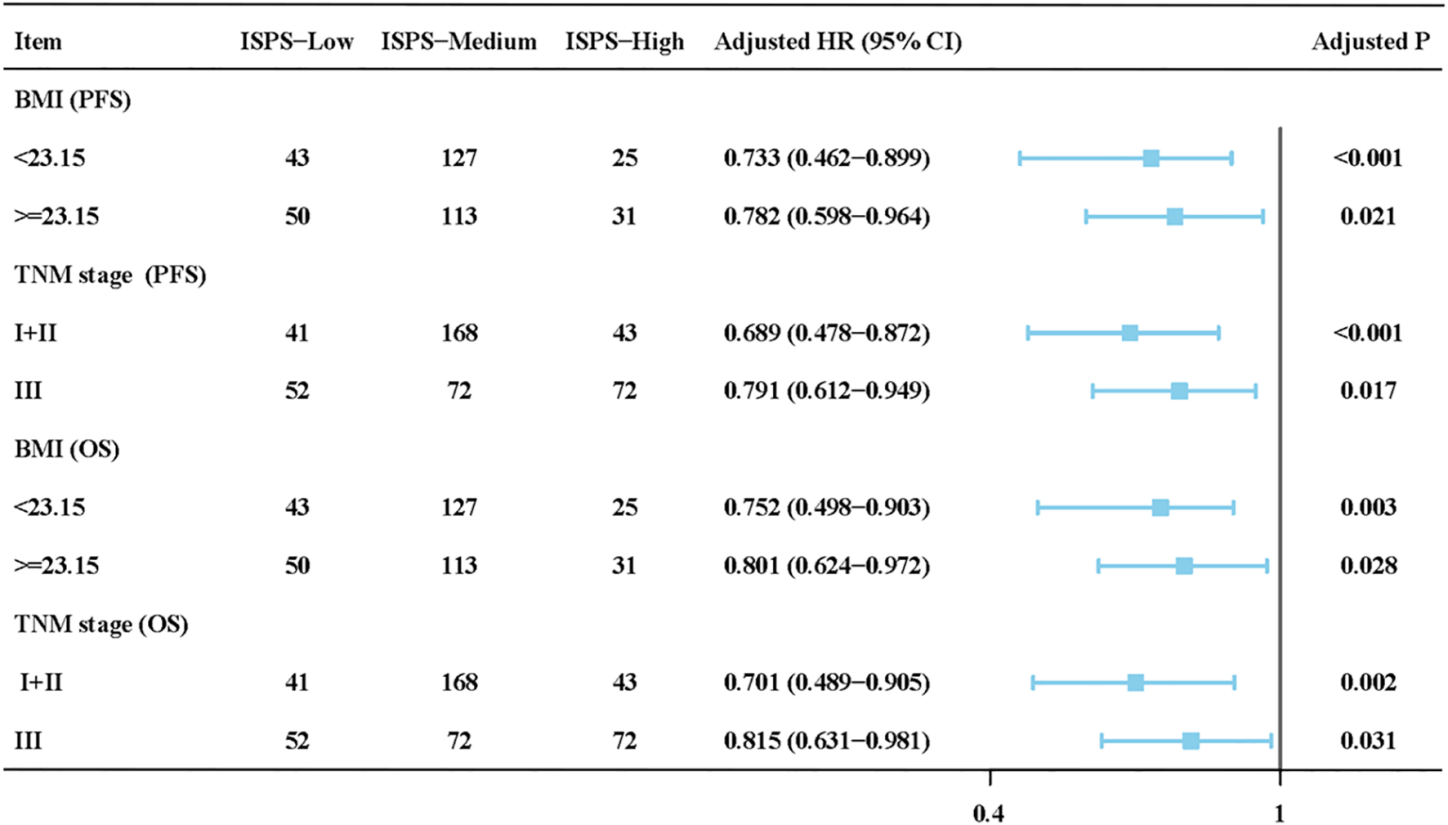
Subgroup and interaction analysis of ISPS for progression-free survival (PFS) and overall survival (OS) stratified by BMI and TNM stage.

To assess whether the prognostic value of ISPS was consistent across clinical subgroups, stratified Cox regression with interaction terms was performed. As shown in **Figure 4**, the associations of ISPS with PFS and OS remained significant across BMI (<23.15 vs. ≥23.15) and TNM stage (I–II vs. III) subgroups. No significant interactions were detected (all P for interaction > 0.1), indicating that the prognostic effect of ISPS was stable across these strata.

### 3.5 Survival analysis of ISPS

**Figure 5:**
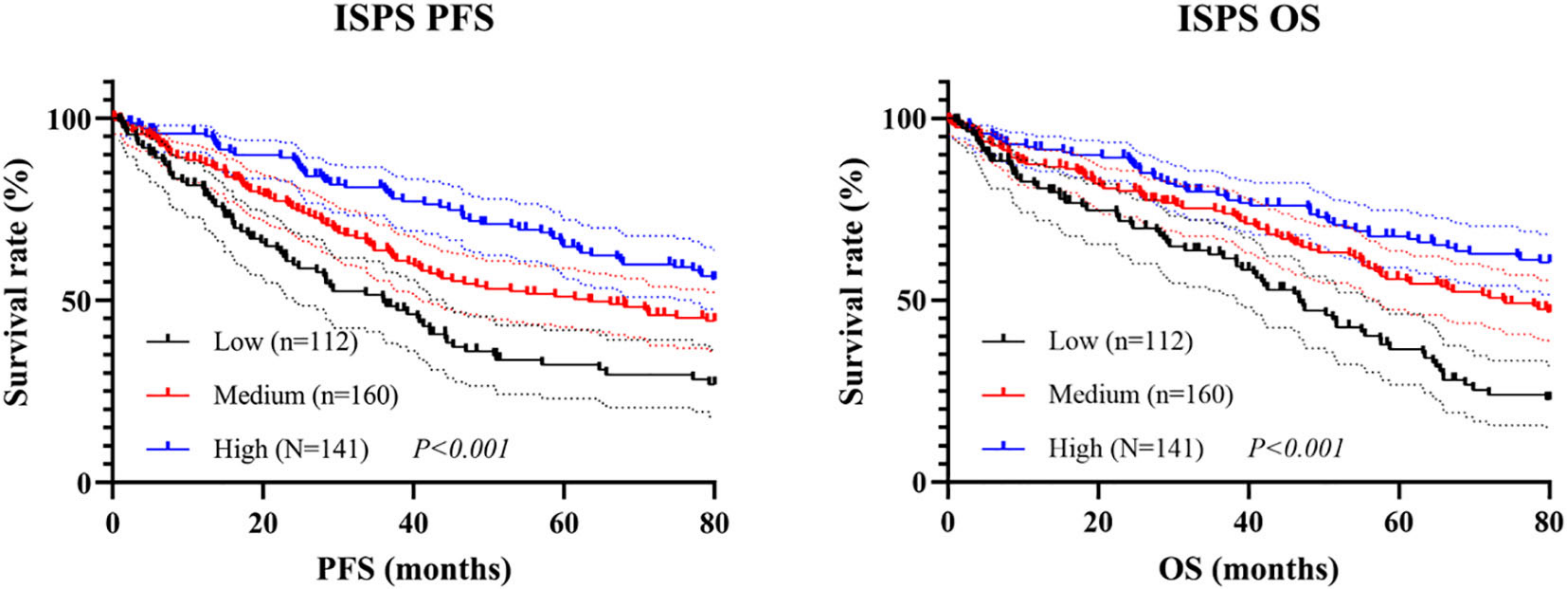
Survival analysis of ISPS in **(A)** PFS and **(B)** OS.

Kaplan–Meier survival analysis was conducted to evaluate the prognostic value of ISPS classification. Patients in the ISPS-High group exhibited the most favorable PFS, followed by those in the ISPS-Medium group, while the ISPS-Low group showed the poorest outcomes. The log-rank test indicated a statistically significant difference among the three groups (χ² = 27.36, P < 0.001, **Figure 5A**), suggesting that lower ISPS scores were associated with earlier disease progression. The median PFS was 38.3 months (95% CI: 32.2–44.7 months) in the ISPS-Low group, 58.6 months (95% CI: 50.1–66.5 months) in the ISPS-Medium group, and not reached in the ISPS-High group. Similarly, for OS, the ISPS-High group demonstrated significantly improved survival compared with the ISPS-Medium and ISPS-Low groups (χ² = 31.54, P < 0.001, **Figure 5B**), further supporting the predictive value of ISPS in long-term clinical outcomes. The median OS was 48.1 months (95% CI: 41.2–55.2 months) in the ISPS-Low group, 75.3 months (95% CI: 65.7–79.7 months) in the ISPS-Medium group, and not reached in the ISPS-High group.

To minimize the potential influence of baseline confounding variables and ensure comparability among groups, PSM was conducted. Patients in the ISPS-Low, ISPS-Medium, and ISPS-High groups were matched in a 1:1:1 ratio (n = 63 per group) using the nearest-neighbor algorithm with a caliper width of 0.02. After matching, no significant differences were observed among the three groups for any of the covariates (all P > 0.05, **Table 6**), indicating adequate baseline balance. To further evaluate balance across matched groups, SMD were calculated for each covariate, with all SMD values < 0.1, confirming acceptable covariate balance after matching.

**Table 6:** Baseline characteristics of ISPS After PSM.

Items	ISPS-Low	ISPS-Medium	ISPS-High	*P* value
	n=63	n=63	n=63	
Age (years), mean (SD)	56.4 ± 8.3	56.0 ± 7.9	55.7 ± 8.4	0.738
Sex (%)				0.891
Male	47 (74.6)	45 (71.4)	46 (73.0)	
Female	16 (25.4)	18 (28.6)	17 (27.0)	
BMI (Kg/m^2^), mean (SD)	20.1 ± 2.3	20.4 ± 2.2	20.3 ± 2.1	0.692
Primary tumor site (%)				0.927
Upper 1/3	9 (14.3)	10 (15.9)	8 (12.7)	
Middle 1/3	20 (31.7)	19 (30.2)	22 (34.9)	
Low 1/3	25 (39.7)	26 (41.3)	24 (38.1)	
Whole	9 (14.3)	8 (12.7)	9 (14.3)	
Tumor size (%)				0.844
<20 mm	10 (15.9)	9 (14.3)	11 (17.5)	
20–50 mm	34 (54.0)	35 (55.6)	33 (52.4)	
>50 mm	19 (30.2)	19 (30.2)	19 (30.2)	
Differentiation (%)				0.624
Poor	26 (41.3)	27 (42.9)	25 (39.7)	
Moderately	21 (33.3)	20 (31.7)	22 (34.9)	
Well	10 (15.9)	11 (17.5)	9 (14.3)	
Unknown	6 (9.5)	5 (7.9)	7 (11.1)	
Lauren type (%)				0.823
Intestinal	36 (57.1)	37 (58.7)	35 (55.6)	
Diffuse	21 (33.3)	20 (31.7)	22 (34.9)	
Mixed	3 (4.8)	4 (6.3)	3 (4.8)	
Unknown	3 (4.8)	2 (3.2)	3 (4.8)	
TNM stage (%)				0.779
I	9 (14.3)	10 (15.9)	11 (17.5)	
II	27 (42.9)	26 (41.3)	25 (39.7)	
III	27 (42.9)	27 (42.9)	27 (42.9)	

**Figure 6:**
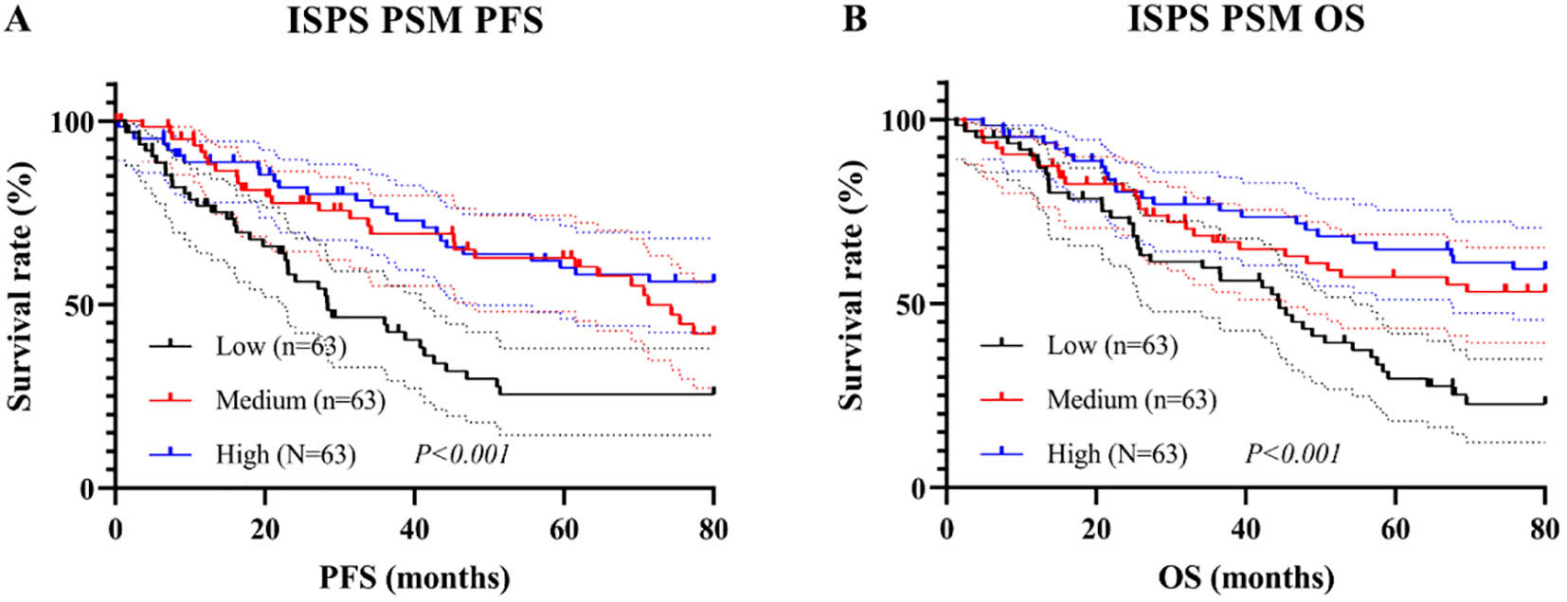
Survival analysis of ISPS in **(A)** PFS and **(B)** OS.

Subsequent Kaplan–Meier analysis after PSM revealed that significant survival differences across ISPS groups persisted. For PFS, patients in the ISPS-High group continued to show the most favorable prognosis, followed by those in the ISPS-Medium group, with the ISPS-Low group exhibiting the poorest outcomes (log-rank χ² = 23.18, P < 0.001; **Figure 6A**). The median PFS was 30.2 months (95% CI: 24.1–36.8 months) in the ISPS-Low group, 72.4 months (95% CI: 61.3–80.5 months) in the ISPS-Medium group, and not reached in the ISPS-High group. Similarly, for OS, the ISPS-High group demonstrated significantly higher survival rates than the other groups (log-rank χ² = 28.36, P < 0.001; **Figure 6B**). The median OS was 48.3 months (95% CI: 40.2–56.7 months) in the ISPS-Low group, while the median OS was not reached in the ISPS-Medium group and the ISPS-High group. These results underscore that higher ISPS scores are robustly associated with improved survival outcomes, even after rigorous adjustment for baseline confounders using PSM.

### 3.6 Survival analysis of CD8^+^ T cell

**Figure 7:**
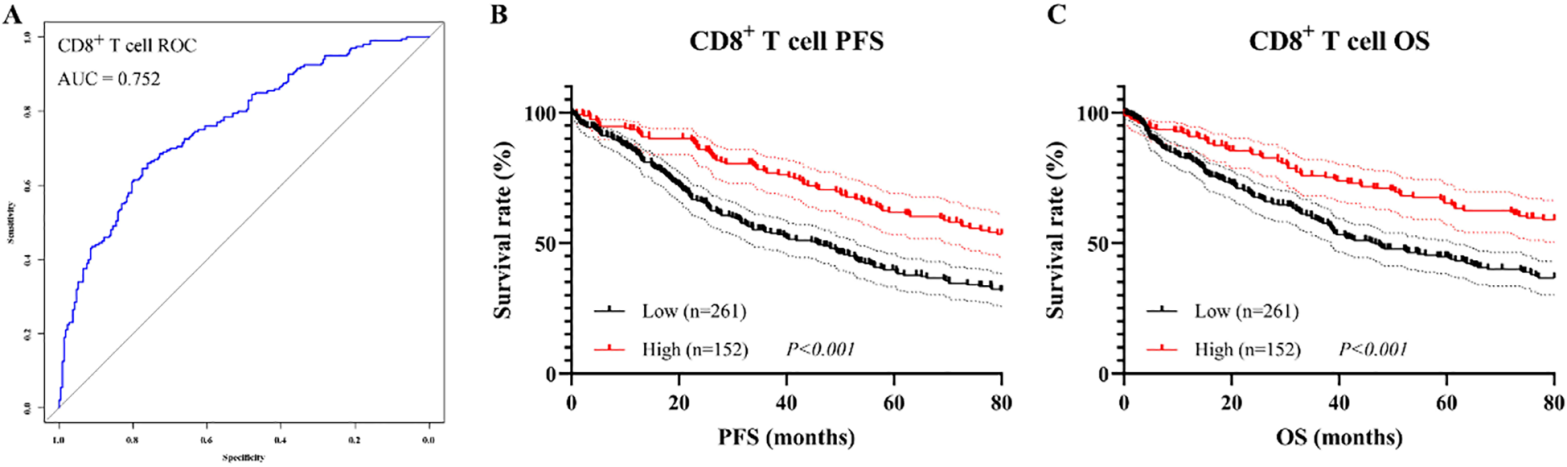
Prognostic value of CD8^+^ T cell proportion before PSM. **(A)** ROC curve for determining the optimal cutoff; **(B)** Kaplan–Meier curve for PFS; **(C)** Kaplan–Meier curve for OS.

As CD8^+^ T cell was identified as an independent prognostic factor in the multivariate Cox regression analysis, survival analyses were subsequently conducted to further investigate its prognostic significance. Based on ROC curve, the optimal cutoff value for CD8^+^ T cell was determined to be 25.3%, with AUC of 0.752, indicating good discriminatory ability (**Figure 7A**). Patients were stratified into CD8^+^ T cell high and low groups accordingly. Kaplan–Meier analysis revealed that patients in the CD8^+^ High group had significantly better PFS and OS than those in the CD8^+^ Low group. Specifically, the log-rank test showed statistical significance for both PFS (χ² = 14.62, P < 0.001, **Figure 7B**) and OS (χ² = 11.23, P < 0.001, **Figure 7C**). The median PFS and OS were 45.3 months (95% CI: 36.8–53.7 months) and 48.8 months (95% CI: 40.5–57.2 months), respectively, in the CD8^+^ Low group, whereas both endpoints were not reached in the CD8^+^ High group.

To reduce baseline confounding and enhance comparability between groups, PSM was conducted using a 1:1 nearest-neighbor algorithm with a caliper of 0.02. After matching, 87 patients were included in each group. No significant differences were observed in any baseline characteristics (all P > 0.05, **Table 7**), and all SMD were < 0.1, indicating adequate covariate balance.

**Table 7:** Baseline characteristics of CD8^+^ T cell After PSM.

Items	CD8^+^-Low	CD8^+^-High	P value
	n = 87	n = 87	
Age (years), mean (SD)	56.9 ± 8.6	55.8 ± 8.4	0.318
Sex (%)			0.412
Male	66 (75.9)	69 (79.3)	
Female	21 (24.1)	18 (20.7)	
BMI (Kg/m²), mean (SD)	20.4 ± 2.1	20.6 ± 2.2	0.572
Primary tumor site (%)			0.698
Upper 1/3	11 (12.6)	13 (14.9)	
Middle 1/3	26 (29.9)	25 (28.7)	
Low 1/3	38 (43.7)	36 (41.4)	
Whole	12 (13.8)	13 (14.9)	
Tumor size (%)			0.466
<20 mm	13 (14.9)	14 (16.1)	
20–50 mm	43 (49.4)	46 (52.9)	
>50 mm	31 (35.6)	27 (31.0)	
Differentiation (%)			0.608
Poor	39 (44.8)	37 (42.5)	
Moderately	26 (29.9)	28 (32.2)	
Well	14 (16.1)	13 (14.9)	
Unknown	8 (9.2)	9 (10.3)	
Lauren type (%)			0.751
Intestinal	36 (41.4)	38 (43.7)	
Diffuse	39 (44.8)	36 (41.4)	
Mixed	6 (6.9)	7 (8.0)	
Unknown	6 (6.9)	6 (6.9)	
TNM stage (%)			0.497
I	9 (10.3)	11 (12.6)	
II	35 (40.2)	33 (37.9)	
III	43 (49.4)	43 (49.4)	

**Figure 8:**
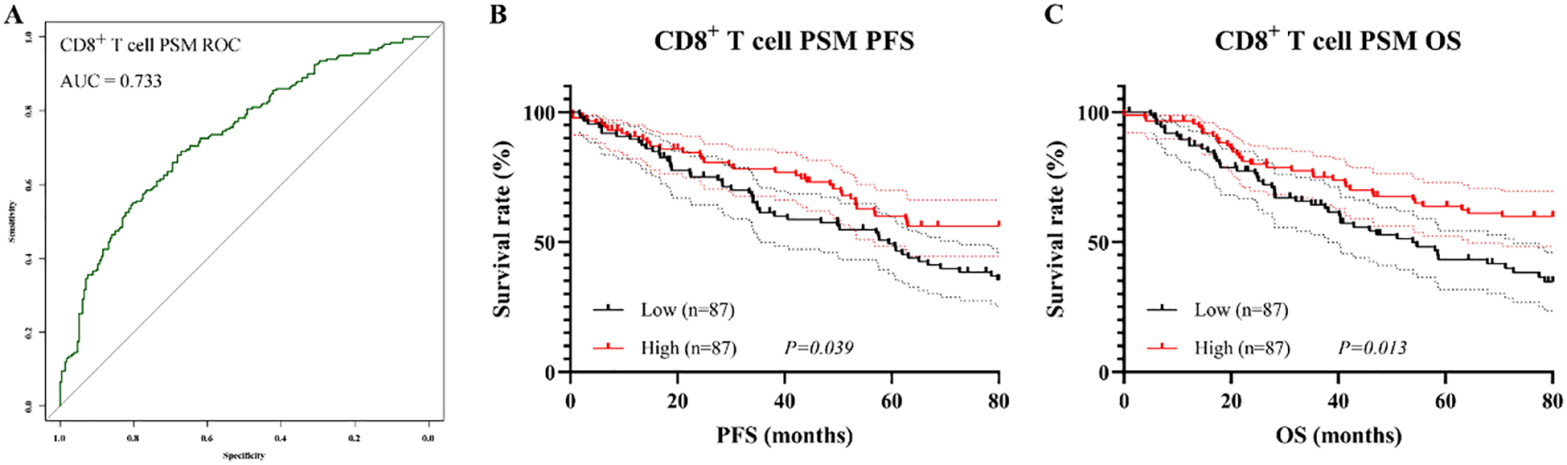
Prognostic value of CD8^+^ T cell proportion after PSM. **(A)** ROC curve after matching; **(B)** Kaplan–Meier curve for PFS; **(C)** Kaplan–Meier curve for OS.

After PSM, ROC analysis yielded an AUC of 0.733, indicating similarly good discriminatory performance (**Figure 8A**). Consistent with the pre-matching results, patients in the CD8^+^ T cell high group continued to exhibit superior survival outcomes compared to those in low group, with significantly longer PFS (χ² = 4.27, P = 0.039, **Figure 8B**) and OS (χ² = 6.12, P = 0.013, **Figure 8C**). The median PFS and OS were 48.2 months (95% CI: 39.5–56.8 months) and 48.5 months (95% CI: 40.1–57.3 months), respectively, in the CD8^+^ Low group, whereas both endpoints were not reached in the CD8^+^ High group.

### 3.7 Mediation analysis

**Figure 9:**
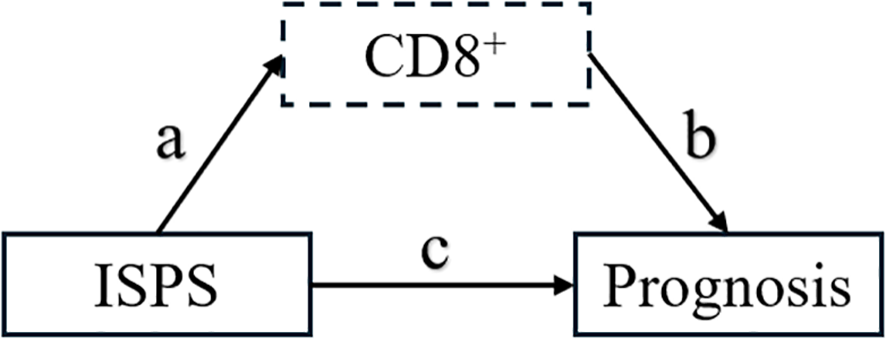
Hypothesized mediation model of CD8^+^ T cell in the relationship between ISPS and prognosis.

To further elucidate the potential immunological mechanism by which ISPS influences patient prognosis, a mediation analysis was conducted using peripheral blood CD8^+^ T cell as the mediator. As shown in the hypothesized mediation model (**Figure 9**), CD8^+^ T cell was posited to transmit part of the prognostic effect of ISPS on survival outcomes.

For PFS, the total effect of ISPS on prognosis was statistically significant (β = –0.378; 95% CI: –0.587 to –0.169; p < 0.001). After adjusting for CD8^+^ T cell, the direct effect remained significant (β = –0.288; 95% CI: –0.490 to –0.086; p = 0.005), while the indirect effect mediated through CD8^+^ T cell was also significant (β = –0.090; 95% CI: –0.170 to –0.030; p = 0.004), accounting for 23.8% (15.2%–32.4%) of the total effect. Similarly, for OS, the total effect of ISPS was significant (β = –0.342; 95% CI: –0.553 to –0.131; p < 0.001), with a direct effect of β = –0.261 (95% CI: –0.470 to –0.052; p = 0.013) and an indirect effect of β = –0.081 (95% CI: –0.140 to –0.025; p = 0.006). The proportion of effect mediated through peripheral CD8^+^ T cell was estimated at 23.7% (14.6%–32.1%) (**Table 8**).

**Table 8:** Mediation analysis results for CD8^+^ T cell.

Effect type	Estimate	95% CI	P	Proportion mediated (95% CI)
PFS				
Total effect	–0.378	–0.587 to –0.169	<0.001	
Direct effect	–0.288	–0.490 to –0.086	0.005	
Indirect effect	–0.090	–0.170 to –0.030	0.004	23.8% (15.2%–32.4%)
OS				
Total effect	–0.342	–0.553 to –0.131	<0.001	
Direct effect	–0.261	–0.470 to –0.052	0.013	
Indirect effect	–0.081	–0.140 to –0.025	0.006	23.7% (14.6%–32.1%)

**Figure 10:**
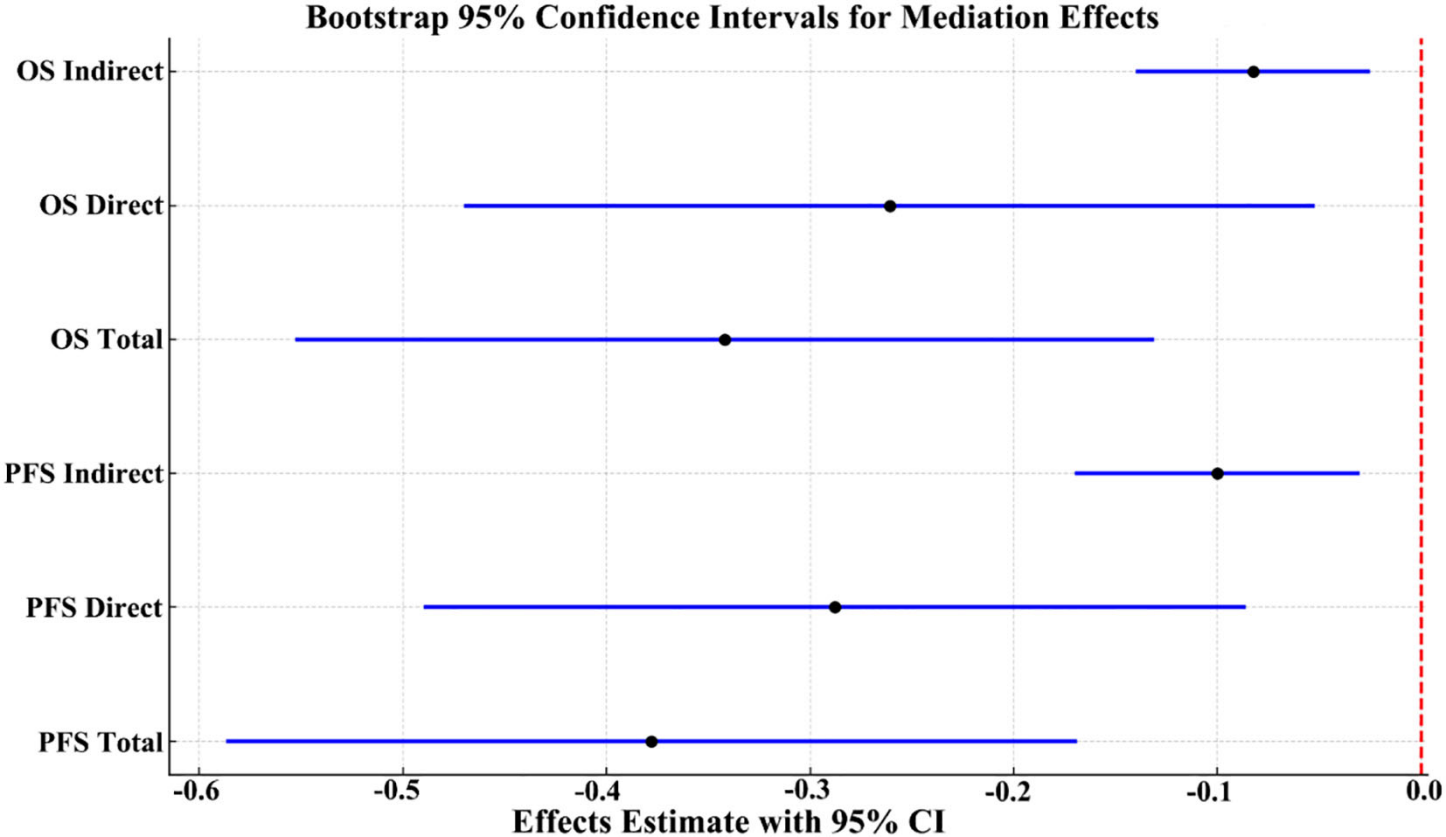
Bootstrap 95% confidence intervals for total, direct, and indirect effects in the mediation model.

To visually confirm the robustness of these findings, bootstrap-based 95% confidence intervals for each pathway were plotted (**Figure 10**), clearly demonstrating statistically significant indirect and direct effects. Collectively, these results support a partial mediating role of circulating CD8^+^ T cell in the association between ISPS and survival outcomes in patients with gastric cancer.

To further verify whether the mediation effect of CD8^+^ T cells was independent of other prognostic determinants, we repeated the mediation analysis after adjusting for postoperative stage, tumor size, age, sex, BMI, and sarcopenia index. As shown in **Table 8**, the total effect of ISPS on prognosis remained statistically significant for both PFS (β = –0.355; 95% CI –0.562 to –0.147; p < 0.001) and OS (β = –0.322; 95% CI –0.529 to –0.112; p < 0.001). After including CD8^+^ T cells in the model, the direct effect of ISPS was slightly attenuated but persisted (PFS: β = –0.276; 95% CI –0.474 to –0.080; p = 0.007; OS: β = –0.245; 95% CI –0.454 to –0.043; p = 0.016). Importantly, the indirect effect mediated through CD8^+^ T cells remained significant (PFS: β = –0.079; 95% CI –0.153 to –0.022; p = 0.006; OS: β = –0.077; 95% CI –0.134 to –0.020; p = 0.008), accounting for 22.30% (14.0%–30.6%) and 21.70% (13.2%–29.8%) of the total effect, respectively. These findings confirm that CD8^+^ T cells consistently mediate part of the ISPS–survival association, even after controlling for stage, tumor size, and sarcopenia, while a direct effect of ISPS beyond the immune pathway remains evident (**Table 9**).

**Table 9:** Covariate-adjusted mediation analysis of CD8^+^ T cells.

Effect type	Estimate	95% CI	P	Proportion mediated (95% CI)
PFS				
Total effect	–0.355	–0.562 to –0.147	<0.001	
Direct effect	–0.276	–0.474 to –0.080	0.007	
Indirect effect	–0.079	–0.153 to –0.022	0.006	22.30% (14.0%–30.6%)
OS				
Total effect	–0.322	–0.529 to –0.112	<0.001	
Direct effect	–0.245	–0.454 to –0.043	0.016	
Indirect effect	–0.077	–0.134 to –0.020	0.008	21.70% (13.2%–29.8%)

### 3.8 Nomograms

**Figure 11:**
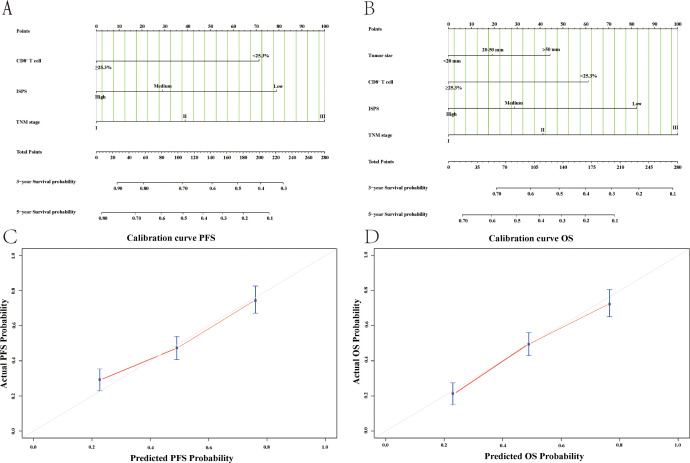
Nomogram models and calibration curves for survival prediction based on the entire study cohort. **(A)** Nomogram for predicting PFS; **(B)** Nomogram for predicting OS; **(C)** Calibration curve of nomogram for the PFS; **(D)** Calibration curve of nomogram for the OS.

To provide an individualized prognostic assessment tool, we constructed two nomograms using the entire study cohort to predict 3-year and 5-year PFS and OS based on independent prognostic factors identified in multivariate analysis. As shown in **Figure 11A, B**, the nomograms incorporated CD8^+^ T cell levels (cutoff at 25.3%), ISPS stratification (high, medium, low), TNM stage (I–III), and tumor size (for OS only). Each variable was assigned a corresponding point value, and the total score could be used to estimate 3-year and 5-year survival probabilities. Calibration plots demonstrated good concordance between the predicted and actual survival outcomes for both PFS (**Figure 11C**) and OS (**Figure 11D**). The C-index for the PFS nomogram was 0.732, while the C-index for the OS nomogram was 0.718, indicating acceptable discriminatory power of both models.

### 3.9 Sample size and power evaluation

To examine whether the study sample size was adequate for multivariable analyses, we conducted a *post-hoc* sample size and power assessment. Among the 413 patients, a total of 232 deaths occurred. The final Cox regression model included 8 parameters, yielding an EPV of 29.0, which exceeds the commonly recommended threshold of ≥10. In addition, based on the Schoenfeld approximation, 232 events provided approximately 80% power (α = 0.05) to detect a hazard ratio of 1.30. These findings indicate that the study sample size was sufficient to support the multivariable Cox regression and mediation analyses.

## 4 Discussion

Growing evidence suggests that sarcopenia may influence cancer progression not only through nutritional deficiency and reduced treatment tolerance but also via its impact on immune function (24). Muscle loss has been associated with chronic inflammation and dysregulated cytokine profiles, which can impair the activity of cytotoxic T cells, particularly CD8^+^ lymphocytes that are essential for antitumor immunity (25). In gastric cancer, this immune impairment may contribute to accelerated disease progression and poorer prognosis. While previous studies have hinted at connections among muscle status, immune suppression, and survival, few have directly examined the potential mediating role of immune parameters. Our findings highlight that reduced physiologic reserve may contribute to worse outcomes in part through diminished CD8^+^ T cell activity, offering mechanistic insight into how sarcopenia and functional impairment translate into immunological vulnerability and adverse clinical trajectories.

In recent years, extensive research has been conducted on the clinical significance of sarcopenia in malignant tumors. Increasing evidence suggests that decreased skeletal muscle mass not only reflects poor nutritional status but is also closely associated with treatment tolerance, immune function, and long-term prognosis. In 2022, Jin and colleagues conducted a systematic review and meta-analysis involving 13 studies and 1,814 patients with ovarian cancer. They found that a low SMI at the L3 vertebral level was significantly associated with shorter PFS and OS, as well as unfavorable clinical characteristics such as lower BMI, more advanced FIGO stage, and reduced likelihood of complete cytoreduction (26). Amitani et al. investigated the prognostic value of SMI changes in patients with breast cancer undergoing neoadjuvant chemotherapy. Their results showed that patients with a decrease in SMI during treatment had significantly worse disease-free survival compared to those with stable or increased SMI, and that SMI loss was an independent risk factor for poor prognosis (27). In a 2024 prospective cohort study conducted in a Chinese population, Chen and colleagues explored the role of sarcopenic obesity (SO) in patients with gastrointestinal cancer. They demonstrated that patients with both sarcopenia and visceral fat accumulation had significantly worse 5-year overall and disease-specific survival, and that SO was an independent predictor of long-term mortality (28). Collectively, these studies further confirm the prognostic value of SMI and its derived indices in various solid tumors, providing important evidence for preoperative assessment and individualized management in cancer patients.

In addition to muscle-related indicators, peripheral lymphocyte subsets have also gained increasing attention as accessible and informative biomarkers for immune status and treatment responsiveness in cancer patients. Liu et al. conducted a retrospective study in patients with advanced non-small cell lung cancer (NSCLC) receiving anti-PD-1 therapy and developed two nomograms incorporating absolute counts of CD3^+^, CD4^+^, CD8^+^, and B cells. Their results demonstrated that lower baseline counts of these lymphocyte subsets were significantly associated with poorer treatment efficacy and shorter progression-free survival, highlighting the prognostic relevance of systemic immune status in the context of immune checkpoint inhibitor therapy (29). Similarly, Zhang and colleagues evaluated the predictive value of lymphocyte subsets and lymphocyte-to-monocyte ratio (LMR) in breast cancer patients undergoing neoadjuvant therapy. They found that higher baseline levels of CD4^+^ T cells, NK cells, and LMR were associated with pathological complete response (pCR), and that dynamic changes in these subsets after treatment further distinguished responders from non-responders (30). In locally advanced esophageal squamous cell carcinoma, Wang et al. reported that patients receiving neoadjuvant immunochemotherapy showed superior pathological response and survival outcomes compared to chemotherapy alone. Their analysis revealed that specific patterns in lymphocyte subsets, such as a lower post-treatment CD4^+^/CD8^+^ ratio, elevated levels of NK cells, and reduced B cell counts, were associated with more favorable immune responses following therapy (31).

In this study, we systematically evaluated the combined impact of sarcopenia and functional impairment, as assessed by skeletal muscle index and ECOG performance status, on immune status and prognosis in patients with resectable gastric cancer. Using the integrated ISPS classification, we found that lower ISPS scores were significantly associated with reduced levels of peripheral CD3^+^, CD8^+^, and NK cells, indicating compromised systemic immunity in patients with poor physical condition. Survival analysis demonstrated that both ISPS and the proportion of CD8^+^ T cells were independent predictors of progression-free and overall survival. Mediation analysis further revealed that CD8^+^ T cells partially explained the association between ISPS and prognosis, accounting for approximately 24 percent of the total effect. Notably, this mediation effect remained robust even after additional adjustment for TNM stage and tumor size, suggesting that ISPS conveys prognostic information that extends beyond classical tumor burden variables. This reinforces the notion that ISPS captures both immune-related and non-immune pathways relevant to patient outcomes. In addition, prognostic nomograms incorporating ISPS, CD8^+^ T cell levels, TNM stage, and tumor size showed good discriminatory ability and calibration, suggesting their potential value in guiding individualized risk assessment. The remaining unexplained proportion of the ISPS–survival association is likely attributable to additional pathways. Potential mediators include compromised nutritional status (e.g., hypoalbuminemia, reduced prealbumin levels), amplified systemic inflammation (e.g., elevated NLR, C-reactive protein, interleukin-6), and tumor-related characteristics such as total burden, metabolic activity, and microenvironmental alterations. These factors may influence both skeletal muscle mass and functional capacity through interconnected metabolic, endocrine, and inflammatory signaling networks, thereby affecting treatment tolerance and long-term survival. Future research incorporating integrated biomarker panels across these domains may provide a more comprehensive mechanistic understanding of the ISPS–survival relationship.

One possible explanation for the prognostic value of the ISPS classification lies in the biological and clinical impact of both sarcopenia and functional impairment. Sarcopenia reflects not only nutritional depletion but also profound alterations in systemic metabolism, hormonal regulation, and inflammatory signaling (32). Loss of skeletal muscle mass has been associated with increased circulating levels of pro-inflammatory cytokines such as interleukin-6 (IL-6) and tumor necrosis factor-alpha (TNF-α), as demonstrated in recent clinical studies of gastric and colorectal cancer patients, where elevated IL-6 and TNF-α were linked to accelerated tumor progression, angiogenesis, and impaired antitumor immunity (33–35). These cytokine-driven inflammatory pathways may also contribute to immune cell exhaustion and reduced treatment tolerance, further explaining the adverse outcomes observed in patients with low ISPS. Meanwhile, impaired physical performance, as indicated by higher ECOG scores, reflects reduced cardiopulmonary reserve, limited activity tolerance, and greater frailty (36). These factors can increase vulnerability to surgical complications, limit the ability to tolerate adjuvant therapy, and contribute to shorter survival (37). Together, sarcopenia and functional impairment provide a comprehensive reflection of reduced physiologic reserve, which is increasingly recognized as a determinant of oncologic outcomes, independent of tumor burden (38). The prognostic role of CD8^+^ T cells in this study is consistent with their well-established function in antitumor immunity. CD8^+^ cytotoxic T lymphocytes are essential for recognizing and eliminating tumor cells through direct cytolytic activity and the release of pro-apoptotic mediators such as perforin and granzyme B (39). High infiltration of CD8^+^ T cells within tumors and robust levels in peripheral blood have been associated with improved survival across multiple cancer types (40). Conversely, reduced CD8^+^ T cell proportions may indicate an exhausted or suppressed immune profile, potentially due to chronic inflammation, malnutrition, or systemic immunosenescence (41). In the perioperative setting, diminished CD8^+^ T cell activity may impair postoperative immune recovery, increase susceptibility to recurrence, and limit the efficacy of immune-based or cytotoxic therapies (42). Therefore, peripheral CD8^+^ T cell levels serve not only as a marker of baseline immune status but also as a modifiable target for optimizing treatment outcomes. Importantly, the partial mediation effect observed in our analysis suggests that the relationship between ISPS and survival is not purely mechanical or metabolic, but also immunologically driven. Patients with low ISPS scores exhibited both functional impairment and decreased CD8^+^ T cell levels, supporting the hypothesis that immune suppression may act as a downstream consequence of poor physical condition (43). Sarcopenia-related inflammation and catabolism may alter T cell differentiation and activation pathways, while reduced physical activity has been associated with impaired immune cell trafficking and function (44). The observed mediation by CD8^+^ T cells thus reveal a plausible biological pathway through which host-related factors such as muscle mass and performance status influence long-term cancer outcomes. This finding provides mechanistic insight into the interplay between the musculoskeletal and immune systems in cancer progression, reinforcing the need for integrative, multidisciplinary approaches to perioperative care and risk assessment.

Notably, the ISPS–CD8^+^-based nomogram not only demonstrated good discriminatory ability and calibration in predicting survival among patients with resectable gastric cancer, but also mechanistically reflects the interplay between structural, functional, and immunological domains. ISPS integrates skeletal muscle mass (as a surrogate for nutritional and metabolic reserve) with ECOG performance status (as an indicator of overall physiological capacity), while CD8^+^ T cell levels directly represent antitumor immune effector function. This multidimensional framework captures systemic status beyond the scope of any single physiological or immune parameter, thereby revealing a plausible biological pathway linking low muscle mass, functional impairment, and immune suppression, and translating it into a practical clinical prediction tool. CD8^+^ T cells are principal effectors of antitumor immunity. They exert direct cytotoxicity through perforin and granzyme B and through engagement of Fas/FasL. Upon activation, they produce IFN-γ and TNF-α, which enhance antigen presentation, increase MHC expression, modulate stromal components, and restrain angiogenesis to strengthen antitumor responses (39, 40). In gastrointestinal malignancies, higher intratumoral CD8^+^ density generally associates with improved survival, whereas phenotypic and functional exhaustion with sustained inhibitory receptor expression and impaired cytokine production correlates with disease progression and treatment resistance (42, 43). These mechanisms provide a biologic basis for our finding that peripheral CD8^+^ T cells partially mediate the association between ISPS and survival and align with the concept that reduced physiologic reserve can coincide with impaired effector T-cell function.

These findings have several important clinical implications. First, the ISPS classification provides a simple, non-invasive, and readily applicable tool for preoperative risk stratification. By integrating skeletal muscle mass derived from routine CT scans with ECOG performance status documented in standard clinical assessments, ISPS captures both structural and functional dimensions of patient fitness. This allows clinicians to identify individuals with reduced physiologic reserve who may benefit from intensified prehabilitation, nutritional support, or closer perioperative monitoring. Second, the incorporation of CD8^+^ T cell profiling offers a valuable immunological perspective in treatment planning. Peripheral immune assessment through flow cytometry is relatively low-cost and feasible in most clinical settings and may help identify patients with immune suppression who are at higher risk of recurrence or poor postoperative recovery. Third, the demonstrated partial mediation effect highlights the potential of targeting immune dysfunction as a modifiable pathway to improve outcomes in physically vulnerable patients. Strategies such as tailored exercise programs, nutritional supplementation, and emerging immunomodulatory interventions may help enhance immune status in the perioperative period. Overall, our study supports the integration of physical and immune evaluations into routine gastric cancer management to facilitate more individualized, comprehensive, and proactive care.

Several limitations of this study should be acknowledged. First, this was a retrospective, single-center study, which may introduce selection bias and limit the generalizability of the findings. Although propensity score matching was applied to reduce baseline imbalances, residual confounding cannot be entirely excluded. Second, the assessment of physical function relied on ECOG performance status, which, despite its clinical practicality, is a subjective measure and may vary depending on the evaluator. More objective tools such as gait speed or handgrip strength could provide additional insights in future studies. Third, although peripheral blood lymphocyte subsets offer a practical surrogate of systemic immune status, they may not fully capture the tumor microenvironment. This study did not evaluate tumor-infiltrating lymphocytes, for example intratumoral CD8^+^ density or tertiary lymphoid structures, which can have prognostic implications. Future studies will include paired blood–tissue analyses with quantitative TIL profiling, for example immunohistochemistry or multiplex immunofluorescence, to delineate the concordance between circulating and intratumoral immunity and to enhance prognostic modeling. Fourth, our mediation analysis assumed a unidirectional pathway in which ISPS influences survival through CD8^+^ T cells. However, the relationship between muscle mass and immune function is likely bidirectional: impaired immunity and chronic inflammation can also promote muscle wasting. Although this modeling choice was based on clinical plausibility and prior evidence, the assumption should be interpreted with caution. Fifth, immune status in cancer patients is known to change dynamically over time, especially after surgery and during adjuvant therapy; because our analysis relied only on preoperative immune data, this may not fully reflect the longitudinal immune trajectory and its impact on survival. Finally, the observational nature of the study precludes causal inference, and the mediation effect of CD8^+^ T cells, although statistically significant, should be interpreted as exploratory. Prospective, multicenter studies with dynamic immune monitoring and intervention arms are warranted to validate and expand upon these findings.

## 5 Conclusion

This study found that sarcopenia and poor physical function are associated with reduced CD8^+^ T cell levels and worse prognosis in patients with resectable gastric cancer. CD8^+^ T cells partially mediate the link between physical status and survival, suggesting that immune dysfunction plays a role in this relationship. These findings highlight the value of combining physical and immune assessments to improve risk stratification and guide personalized care. Moreover, ISPS may serve as a useful prognostic tool, although further multicenter and prospective studies are needed to validate its clinical applicability.

## Data Availability

The raw data supporting the conclusions of this article will be made available by the authors, without undue reservation.
